# Technological, environmental and biological factors: referent variance values for infrared imaging of the bovine

**DOI:** 10.1186/s40104-015-0027-y

**Published:** 2015-06-12

**Authors:** Yuri R. Montanholi, Melissa Lim, Alaina Macdonald, Brock A. Smith, Christy Goldhawk, Karen Schwartzkopf-Genswein, Stephen P. Miller

**Affiliations:** Department of Animal and Poultry Science, University of Guelph, Guelph, ON N1G 2W1 Canada; Department of Plant and Animal Sciences, Dalhousie University, Truro, NS B2N 5E3 Canada; Monsanto, Headingley, MB R3T 6E3 Canada; Agriculture and Agri-Food Canada, Lethbridge, AB T1J 4B1 Canada; Faculty of Veterinary Medicine, University of Calgary, Calgary, AB T2N 4Z6 Canada; Invermay Agricultural Centre, AgResearch Limited, Mosgiel, 9053 New Zealand

**Keywords:** Body heat loss, Convective heat loss, Infrared imaging, Oxygen consumption, Pharmacodynamics

## Abstract

**Background:**

Despite its variety of potential applications, the wide implementation of infrared technology in cattle production faces technical, environmental and biological challenges similar to other indicators of metabolic state. Nine trials, divided into three classes (technological, environmental and biological factors) were conducted to illustrate the influence of these factors on body surface temperature assessed through infrared imaging.

**Results:**

Evaluation of technological factors indicated the following: measurements of body temperatures were strongly repeatable when taken within 10 s; appropriateness of differing infrared camera technologies was influenced by distance to the target; and results were consistent when analysis of thermographs was compared between judges. Evaluation of environmental factors illustrated that wind and debris caused decreases in body surface temperatures without affecting metabolic rate; additionally, body surface temperature increased due to sunlight but returned to baseline values within minutes of shade exposure. Examination/investigation/exploration of animal factors demonstrated that exercise caused an increase in body surface temperature and metabolic rate. Administration of sedative and anti-sedative caused changes on body surface temperature and metabolic rate, and during late pregnancy a foetal thermal imprint was visible through abdominal infrared imaging.

**Conclusion:**

The above factors should be considered in order to standardize operational procedures for taking thermographs, thereby optimizing the use of such technology in cattle operations.

## Background

Infrared radiation refers to radiation that has a longer wavelength than visible light. Its history began in 1800, when Sir William Herschel discovered heat rays that went beyond the scope of the visible red colour in the electromagnetic spectrum [1]. This discovery culminated in the development of several infrared radiation based applications, including infrared imaging. Infrared imaging technology, which is noted for its non-contact operation, has evolved from its initial military use in the 1950’s to a multitude of applications, including several in the study of wild and domestic animals. In wild animals this technology has been used to determine dorsal fin surface temperatures of dolphins [2], optimize the counts of white tailed deer via aerial appraisal [3], evaluate heat exchange patterns in African elephants [4], and determine metabolic heat loss in birds [5].

A comprehensive collection of infrared imaging applications in livestock animals is described by Luzi et al. [6]. Applications in the livestock sector, particularly in bovine operations, include early detection of inflammatory illness including mastitis [7], lameness [8] and bovine respiratory disease virus [9]; assessment of animal welfare issues such as tail docking sensitivity [10] and reactivity to stress [11]; determination of the appropriateness of milking equipment [12]; assessment of productivity, including of heat and methane production [13] and feed efficiency [14]; evaluation of fertility traits, such as scrotum temperature patterns [15] and body condition score [16]; and appraisal of meat quality [17].

Despite the myriad of potential applications, the use of infrared imaging in commercial cattle husbandry is not widespread. There is, however, great potential for expansion as reviewed by Mitchell [18]. As with other highly sensitive measures, radiant heat loss poses challenges related to adequacy of equipment, as well as environmental and biological factors that can alter thermograph readings. The choice of the infrared camera based on the available technological options should be considered [19]. Environmental and animal factors may mask the underlying biological target by causing uncontrolled changes in the patterns of radiation detected by the infrared camera. In the case of livestock species, infrared imaging detects small changes in body surface temperature patterns caused by biological phenomena [11, 13], making the infrared imaging assessment subject to artifacts. Environmental factors include debris covering the animal which blocks the normal infrared radiation emissions from the body surface. Additionally, weather conditions such as sunlight exposure and air movement [20] can also skew infrared analyses by interfering with the radiation emitted by the animal’s body surface. A few examples of biological effects on metabolic rate, and consequently body temperature, include physical activity [21], physiological states such as pregnancy [22], and pharmacodynamics [23].

Since infrared radiation emissions captured from animals through infrared images may be largely influenced by several factors, it is important to characterize and consider these potential effects to ensure reliable and accurate infrared imaging assessments. Our objectives were several fold: a) to compare the types of infrared technology and measure the consistency of imaging analysis; b) to demonstrate the effects of environmental artifacts (wind, debris on body surface, and sunlight exposure) on infrared imaging; and c) to illustrate the effects of biological factors (physical exercise, drug response, and pregnancy) on thermographs.

## Methods

### General information about research trials and animals

The study was divided into three classes with a total of nine research trials. The classes included the following: (1) technological factors (imaging repeatability, infrared camera comparison, and judge comparison trials); (2) environmental factors (wind, debris on body surface, and sunlight exposure); and (3) biological factors (physical exercise, drug response, and pregnancy status). Trials were conducted at the Elora Beef and Dairy Research Centres (University of Guelph). The animal care protocol and experimental procedures in this study were approved by the University of Guelph Animal Care Committee. A number of animals (2 beef heifers, 128 beef calves, 3 beef cows and 1 dairy cow) were used to accomplish the different trials, as detailed in Table 1. For the physical exercise, sedative response, wind, and debris on body surface trials, an indirect calorimeter based on gas exchange, similar to the system described by Odongo et al. [24], was used to assess real-time metabolic rate with measures of oxygen consumption obtained every 1 s during sampling. For infrared camera comparison, wind, debris on body surface, physical exercise, drug response and pregnancy status trials, temperature and humidity were monitored every 5 min (Hobo Pro U14, U.S.A.). Additionally, a shaved patch of 5 by 5 cm, located at the dorso-caudal portion of the flank, was made on the beef heifers for skin surface assessment. Assessment of respiration rate during the physical exercise and sunlight trials was performed by counting flank movements over a period of 1 min during each assessment, in accordance with the method previously described by Gaughan et al. [25].

**Table 1:** Description of the animals used over the different research trial

Animals	Age, d	Weight, kg	Status	Breed composition
Beef heifer A	487	496	Non-pregnant	50 % Piedmontese, 35 % Angus 15 % Simmental,
Beef heifer B	453	492	Non-pregnant	50 % Piedmontese, 50 % Simmental
128 Calves	194 ± 21	260 ± 40.2	Weaned	45 % Angus, 40 % Simmental, 15 % Piedmontese;
3 Beef cows	5079 ± 440	689 ± 63.7	Post-partum, nursing	33 % Angus, 33 % Piedmontese, 17 % Simmental, 17 % other breeds
Dairy heifer	738	560	Pre-calving	100 % Holstein

### Infrared imaging and image analysis

**Fig. 1:**
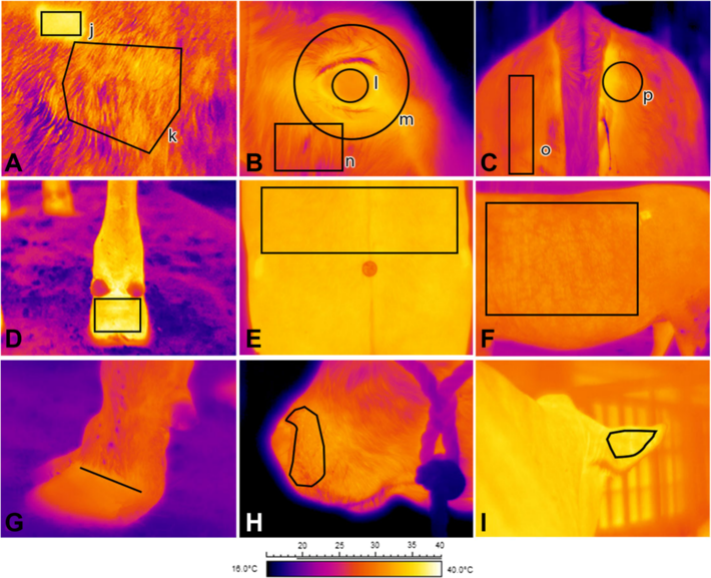
Infrared images of the body locations evaluated throughout the trials; (**a**): flank (*k*), shaved patch (*j*), (**b**): eyeball (*l*), whole eye (*m*), cheek area (*n*), (**c**): hind area using a rectangle (*o*), hind area using a circle (*p*), (**d**): rear view of the foot, (**e**): lumbar region, (**f**): trunk, (**g**): coronary band, (**h**) snout, (**i**) rear view of the ear. The shapes drawn on the images delimit the portion of the image used to access the temperature during imaging analyses

Infrared images from different body locations were taken (Fig. 1) with one or all of the following infrared cameras: FLIR i40, FLIR T250 and FLIR SC2000 (FLIR Systems, Inc., U.S.A.). The FLIR i40 represents the simplest technology, with a temperature range of −20 to 350 °C, and resolution of 120 × 120 pixels. The FLIR T250 represents technology of intermediate quality, with a temperature range of −20 to 350 °C, and a resolution of 240 × 180 pixels. The FLIR SC2000 is of scientific grade, has a temperature range of −40 to 1500 °C, a resolution of 320 × 240 pixels, and also multiple options for lenses (12, 24 and 45° lenses).

Analyses of the thermographs were performed using the ThermaCAM Researcher Pro 2.8 SR-1 software (FLIR Systems, Inc., U.S.A.). The emissivity value was set at 0.98 for all infrared imaging analyses. Images were captured and analyzed using the iron palette for all trials except for the pregnancy status trial, where the rain 10 palette was used. Both the iron and rain 10 palettes display black and white colors indicating the coldest and warmest temperatures respectively, with other colors indicating intermediary temperatures. For the iron palette the color pattern is continuous from pixel to pixel, while in the rain 10 palette the whole temperature distribution of the image is divided in ten ranges with each receiving a different color. After an appropriate shape was drawn on each image to outline the body sub-location, the maximum and average temperatures of the selected region were computed (Fig. 1). The shapes were determined based on anatomical landmarks as well as biological relevance for body temperature control [26], and were based on previous studies on body surface temperature assessment using infrared imaging in the bovine [11, 13].

### Technological factors

#### Imaging repeatability trial

The imaging repeatability trial used beef calves (Table 1). Calves were divided into two groups (with 69 calves in the first group and 59 calves in the second), and groups were sampled on two consecutive weeks. Each individual animal was infrared imaged and its body weight assessed while calves were restrained in a squeeze chute (Silencer Hydraulic Squeeze Chute; Moly Manufacturing Inc., U.S.A.) located indoors. Two head thermographs were taken consecutively (within 10 s) with the FLIR SC2000 camera. All calves were submitted to the same sampling regime 72 h later. The images were taken between 1400 and 1600 h. The repeatability of the eye and the snout analysis, for both average and maximum temperatures, was then accessed (Fig. 1; bm, h).

#### Distance and infrared cameras comparison trial

Images were taken of the shaved square patch located on the left flank (Fig. 1; aj) of the beef heifer A (Table 1). Images were taken from seven distances (1.0, 1.5, 3.5, 5.5, 9.5, 11.5 and 15.5 m) with the three infrared cameras, and with the three different lenses for the SC2000 camera. The heifer was tied to a post indoors using a halter and allowed to acclimatize to the environment before the imaging procedure. All images were taken within 40 min with the heifer standing still.

#### Judge comparison trial

Two trained judges who were experienced with infrared imaging interpretation analyzed the images, which were all taken 1 m from the animal surface. Images of the foot, eye, snout and hind area, as well as the images of the shaved square patch were taken for use in this trial. Body surface landmarks were established beforehand to ensure consistency between temperature readings from the infrared images among the two judges. For the flank, an irregular hexagon was drawn following the anatomical landmarks delimiting the flank region (Fig. 1; ak). The foot was analyzed via a rectangle placed below the dew claw and immediately above the hoof (Fig. 1; d). The coronary band temperature was measured via a line drawn at the lateral view of the outer hoof (Fig. 1; g). To measure eye temperature, a circle was drawn to cover the entire eye and surrounding area; the diameter of the circle was equal to the width of the eye plus distance between the eye medial canthus and the end of the warmest region medial to the canthus (Fig. 1; bm). A polygon spanning the lower edge of the nostril and across the top of the upper lip was created to analyze the snout (Fig. 1; h). Lastly, the hind area was analyzed using a circle with a diameter equal to the width of the tail at its insertion and placed immediately dorsolateral to the vulva (Fig. 1; cp).

### Environmental factors

#### Wind trial

Heifer B (Table 1) was used in this trial and infrared images were taken using the FLIR SC2000 camera with the 45° lens at a distance of 0.8 m. Images were taken of the hind foot, trunk, flank and clipped square on both sides of the animal (Fig. 1; d, f, ak and aj). A set of baseline images were taken, and 20 min later a fan (Protemp PT-36-BDF-AF, Pinnacle Products International, Inc., U.S.A.) was turned on at a wind speed of 17 km/h, measured using an anemometer (La Crosse Technology EA-3010U, U.S.A.). The wind was directed at the left side of the animal from a distance of 0.9 m from the trunk of the heifer, while the head of the animal was held in the calorimetric chamber. Images were taken as soon as the fan was turned on and every 20 min thereafter, for a total of four sets. The fan was then turned off and images were taken every 20 min for a total of three additional sets.

#### Debris on body surface trial

Heifer B (Table 1) was used in this trial and infrared images were taken using the FLIR SC2000 camera with the 45° lens at a distance of 0.8 m. The debris on body surface trial was conducted by placing dried wood shavings on the back while the head of the animal was held in the indirect calorimeter. Unlike the other trials, only one specific body location, a top view of the lumbar region, was the focus for this trial (Fig. 1; e). One image was taken as a baseline every 20 min for a total of four images, with the fourth image taken immediately before adding shavings (cooled to 10 °C) on top of the animal’s back, and a fifth image being taken immediately after the shavings were put on. Shavings remained on the animal for 40 min, with one image taken every 20 min for a total of two images. Shavings were then removed from the animal with a soft brush and a final image was taken 20 min later under the same conditions as the previous images.

#### Sunlight exposure trial

A sunlight exposure trial was conducted using three beef cows (Table 1). Infrared images were taken using the FLIR SC2000 camera with the 24° lens at a distance of 1.0 to 1.5 m from the object. Images were taken from the flank, the hind area, the eyeball, the snout and the back of the ear (Fig. 1; ak, co, bl, h, i). The solar radiation was measured by a light meter (Li-Cor LI-189, U.S.A.). The temperature and relative humidity were assessed using a thermometer and humidity data logger (Hobo Pro v2 U23-002, U.S.A.), and wind speed was measured using an anemometer (Sims DIC-3, Simerl Instruments, U.S.A.). An adapted heart rate monitor (Polar WearLink W.I.N.D. transmitter, Polar Electro Oy, U.S.A.) was attached to the animal via a leather belt strapped around the girth of the animal, and respiration frequency assessed as previously described every 7 min for a total of 15 times. Measurements of heart rate and environmental temperature were taken every 5 and 60 s respectively, and averaged over 7 min intervals for the entire duration of the trial. Cows were housed in individual and neighboring pens and were supervised by one herdsman per cow to ensure minimal physical activity during this trial. Cows were initially confined to a shaded area for 30 min of standing with minimum physical activity; this was then followed by a set of baseline images taken for a total of three sets every 7 min. The three cows were then moved and kept in a sunny area, where a set of images was taken every 7 min for a total of six sets, before being led back to the shaded area where another set of images were taken every 7 min for a total of six sets.

### Biological factors

#### Physical exercise trial

Heifer A (Table 1) was used in this trial and infrared images were taken using the T250 camera at a distance of 0.8 m. Information was gathered for heat production with images taken of the clipped left flank area, the front foot, eyeball, cheek and snout (Fig. 1; aj, d, bn and h). Following three sets of baseline images and concurrent metabolic rate assessments, the heifer was then subjected to 15 min of exercise which consisted of chasing the heifer to run across a covered barn of 80 m in length. The entire exercise portion took a total of 20 min; 15 min for the exercise trial and 5 min to set the heifer back up in the calorimeter). A later set of post-exercise images and calorimetry assessments were taken every 5 min after exercising for three sets. Respiration rate was also assessed during the baseline and post-exercise periods.

#### Drug response trial

The drug response trial was separated into two portions. Heifer B was used in the first portion and Heifer A was used in the second portion (Table 1). Infrared images were taken from the right flank, the coronary band, the foot, and the hind area (Fig. 1; ak, g, d, co). In the first portion, where only xylazine (Rompun, Bayer) was utilized, baseline images were taken every 7.5 min for three sets with the FLIR SC2000 camera at a distance of 0.8 m. The animal was then injected with 0.02 mg/kg of xylazine (Rompun, Bayer) intravenously to obtain mild sedation while maintained in the calorimetric chamber, and a subsequent set of images were taken after this injection every 7.5 min for a total of 12 sets. The second portion of the trial involved xylazine and atipamezole (Antisedan, Pfizer), with xylazine first administered to the animal (0.02 mg/kg; IV), followed by atipamezole (0.10 mg/kg; IV) 40 min after the xylazine. A set of baseline images was taken every 20 min for three sets, followed by the xylazine injection. A set of images was then taken every 20 min following the xylazine injection for two more sets, and after injection of atipamezole, two additional sets of images were taken every 20 min. Calorimetry measures were taken for the animals throughout the trial, with information gathered for heat production.

#### Pregnancy status trial

A pregnant dairy heifer was imaged for this trial (Table 1) using the SC2000 camera, equipped with the 45° lens, at a distance of 4.0 m. The heifer was housed indoors in an individual free-stall where the images were also taken. Infrared images were taken of the left and right sides of the trunk of the animal (Fig. 9), upon ensuring the heifer was standing for 30 min and had a dry hair coat free of debris.

### Data analysis

Descriptive statistics (average, standard deviation, maximum and minimum values) and Pearson correlation analysis for all the nine trials, except pregnancy status, were conducted using the means and correlation procedures of the statistical analysis system (version 9.1, SAS Institute, U.S.A.). The mean procedure was employed on the dataset of the following trials: physical exercise, drug response, wind, debris on body surface, and sunlight exposure; in order to calculate the descriptive statistics of the distinct events within each of these trials (i.e. baseline readings and experimental treatment responses). For the sunlight exposure trial, the average across different cows was calculated. Additionally, in the infrared camera comparison trial, means of all of equipment used were calculated at each distance. For the imaging repeatability trial, correlations between images taken consecutively and across different days, as well as between body weight assessed on different days, were determined. Similarly, correlations between judges across different body locations and by determinations of the average or maximum temperature were also calculated.

## Results

### Technological factors

#### Imaging repeatability trial

Table 2 shows the infrared imaging results of the repeatability trial. For the four days of assessment (with two days for each group of calves), strong correlations were observed between images taken consecutively at both body locations (snout and eye) and for both analyses (average and maximum temperatures). Correlations between days for both groups were weak, with average temperatures for both locations displaying better repeatability than maximum temperatures (Table 2). Average temperatures also presented more consistent correlations between the two groups over maximum temperatures. A strong correlation between body weights of animals at the two different weight assessments was also observed for both groups; correlations were 0.97 and 0.89 for groups 1 and 2, respectively (*P* < 0.01).

**Table 2:** Correlations between infrared imaging measures (average and maximum temperatures) taken from snout and eye^a^

Group	Comparison	Snout		Eye	
		Maximum	Average	Maximum	Average
Group 1	Images 1 and 2 (day 1)	0.97*	0.99*	0.95*	0.96*
	Images 1 and 2 (day 2)	0.91*	0.99*	0.82*	0.97*
	Average day 1 and 2	0.28**	0.34*	0.13	0.37*
Group 2	Image 1 and 2 (day 1)	0.76*	0.98*	0.79*	0.96*
	Image 1 and 2 (day 2)	0.91*	0.93*	0.87*	0.95*
	Average day 1 and 2	0.16	0.31**	0.22	0.45*

*P < 0.05; ** 0.05 < *P* ≤ 0.10

^a^Images were taken from two groups of calves (Group 1 and Group 2) on two different days (Day 1 and Day 2), with two consecutive images in each day (Image 1 and Image 2)

#### Distance and infrared cameras comparison trial

**Fig. 2:**
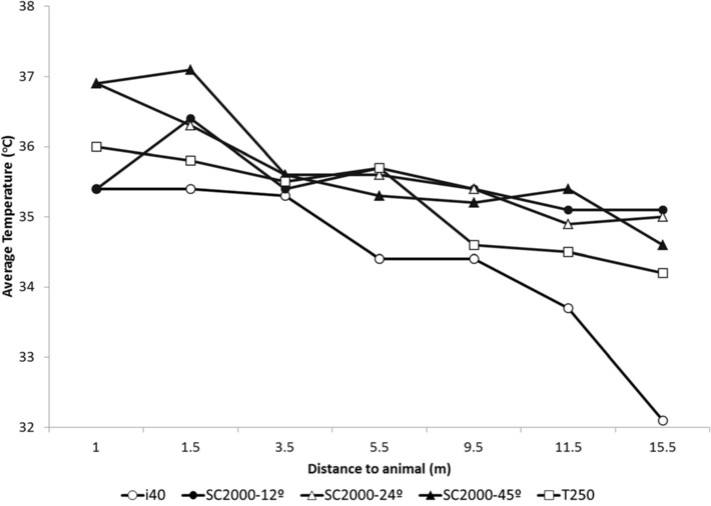
Infrared imaging measures by different camera types and lenses and at different distances from the flank clipped area

The average relative humidity was 69.8 ± 1.7 % and the average temperature was 22.9 ± 0.5 °C for the duration of the infrared cameras comparison trial. Figure 2 shows the temperature of the shaven patch obtained using five infrared imaging technologies from seven distances. The overall mean ± SD, for each technology and across different distances, for the i40, T250, SC2000-12°, SC2000-24° and SC2000-45° was 34.4 ± 1.2, 35.2 ± 0.7, 35.5 ± 0.5, 35.7 ± 0.7, and 35.7 ± 0.9 °C respectively. The overall mean ± SD for each distance, namely 1.0, 1.5, 3.5, 5.5, 9.5, 11.5 and 15.5 m from the targeted body location was 36.1 ± 0.7, 36.2 ± 0.6, 35.5 ± 0.1, 35.3 ± 0.6, 35.0 ± 0.7, 34.7 ± 0.8 and 34.2 ± 1.4.

#### Judge comparison trial

**Fig. 3:**
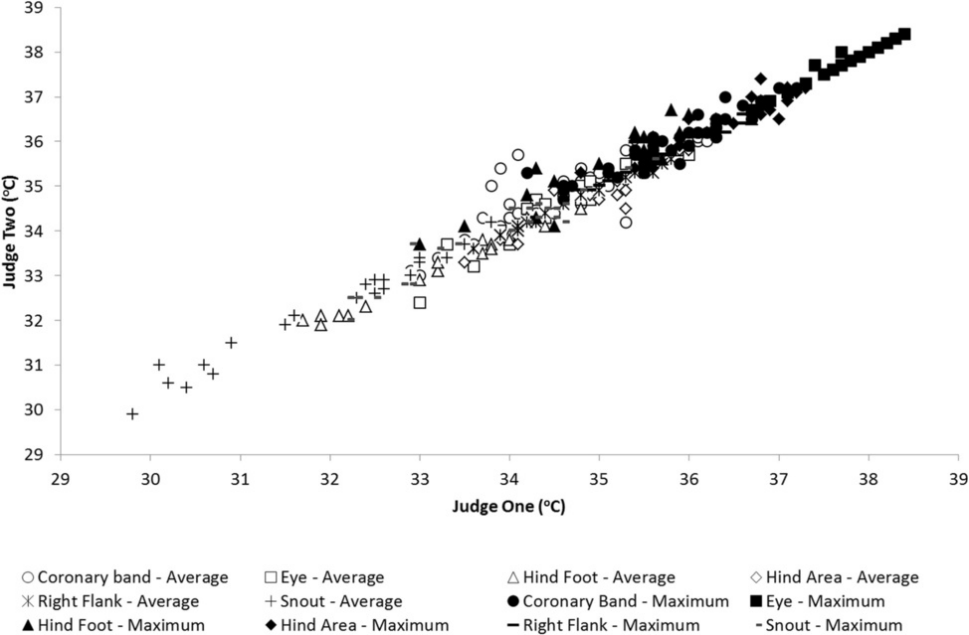
Infrared imaging measures by two judges for maximum (*solid shapes*) and average (*open shapes*) temperatures at six body locations

Results indicate a strong association between the infrared imaging results obtained by each of the two judges, with a correlation of 0.96 and 0.98 (*P* < 0.05) between the judges for the average and maximum temperatures respectively, across the six body locations studied (coronary band, eye, hind foot, hind area, right flank and snout) (Fig. 3).

### Environmental factors

#### Wind trial

**Fig. 4:**
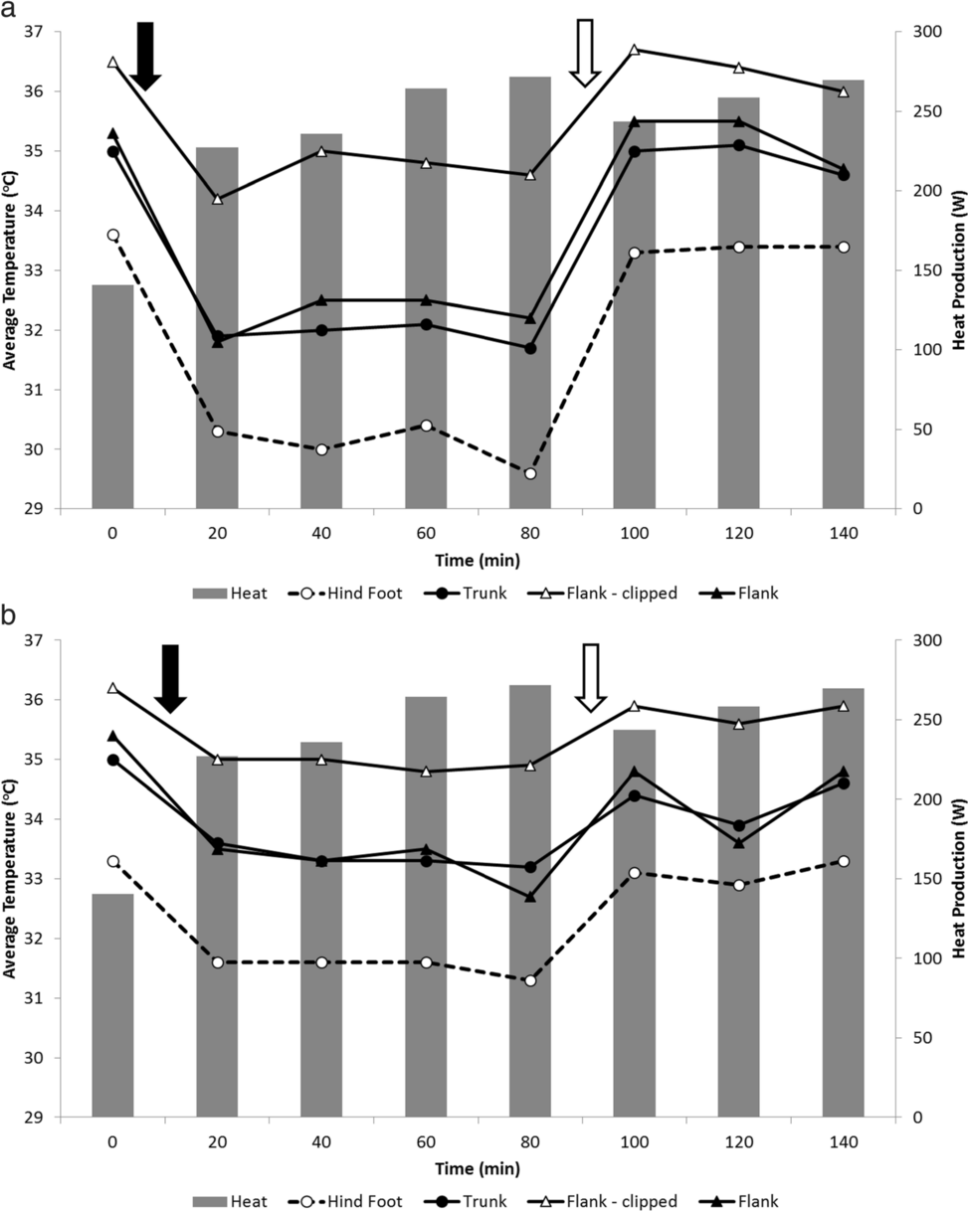
Infrared imaging measures and heat production in response to wind. (**a**) left and (**b**) right sides of the animal in response to a wind being blown on the left side of the animal. The **solid** and *open arrows* denote the fan being turned on and off

The average relative humidity was 62.8 ± 2.2 % and the average temperature was 25.4 ± 0.3 °C for the duration of the wind trial. The wind, directed to the left side of the animal, caused an immediate and abrupt decrease in the body surface temperature at the four body locations evaluated (Fig. 4a). A similar response was also observed on the right side, but of a lower magnitude (Fig. 4b). The temperature of the left and right trunk decreased from 36.5 to 31.9 °C and 36.2 to 33.4 °C, respectively, in response to the wind. Figure 4 illustrates a lack of association between heat production and infrared traits during the period that the animal was subjected to wind on its left side, and a re-establishment of such association after the fan was turned off.

#### Debris on body surface trial

**Fig. 5:**
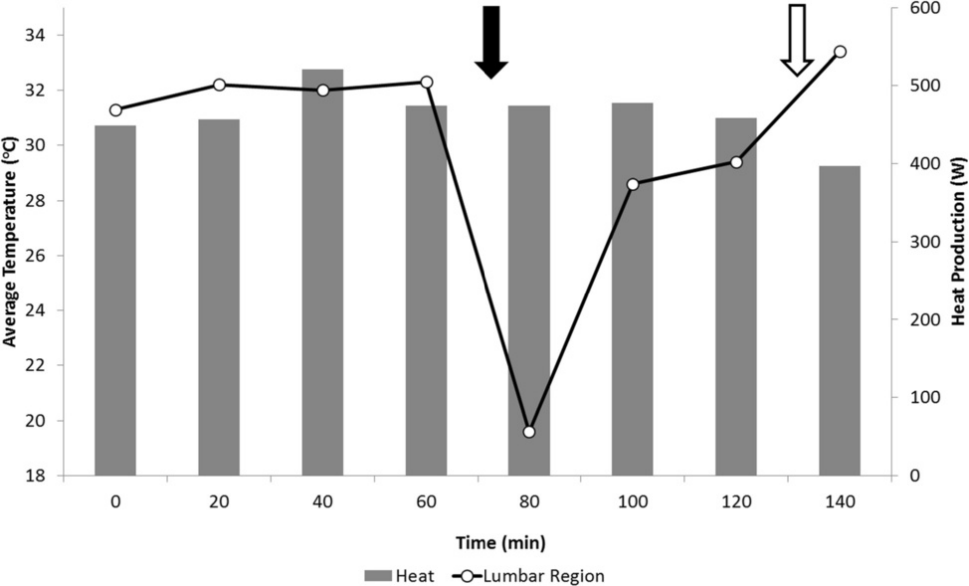
Infrared imaging measures and heat production in response to shavings partially covering the animal skin surface. The *solid* and *open arrows* denote the spread and removal of shavings on the lumbar region of the animal

The average relative humidity was 79.1 ± 1.6 % and the average temperature was 22.7 ± 0.4 °C during evaluation of the effects of debris on the body surface. The four sets of infrared images and heat production data collected prior to adding shavings indicate a similarity between the values of these two measures (Fig. 5). Immediately after adding shavings, heat production was unaffected but the temperature of the lumbar region drastically decreased from 32.3 to 19.0 °C (Fig. 5). The temperature of the lumbar region subsequently rose to 28.6 °C after 20 min, and continued to increase at a slower pace until 40 min after when the shavings were brushed off. After brushing, the temperature of the lumbar region was the highest observed throughout the entire trial, and showed a significant mismatch with heat production (Fig. 5).

#### Sunlight exposure trial

**Fig. 6:**
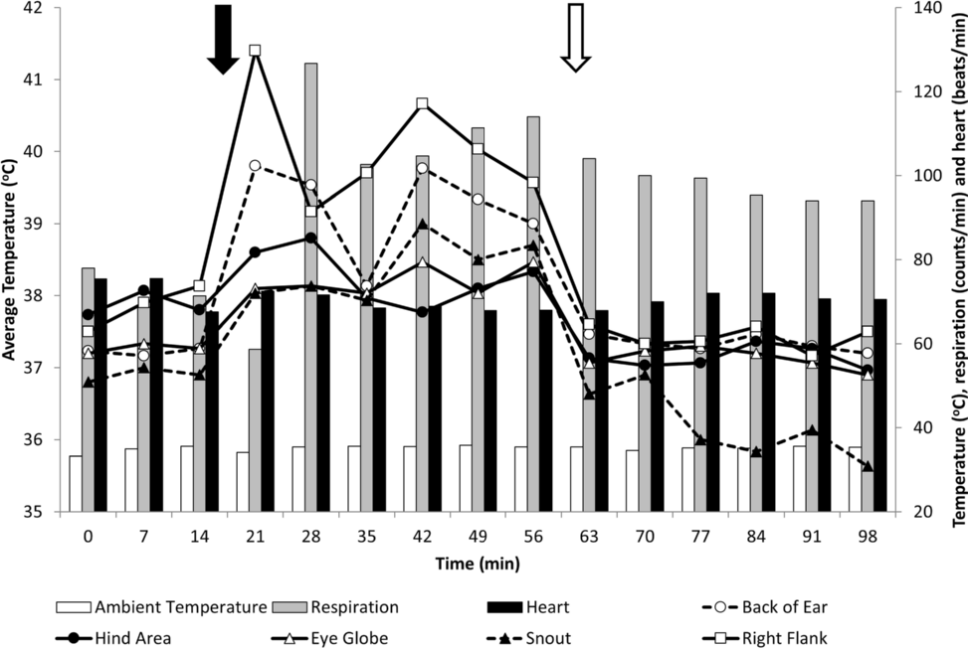
Infrared imaging measures, ambient temperature, heart and respiration rates of beef cows in response to sun or shade exposure. The *solid* and *open arrows* denote the start and end of the sun light exposure

During the sunlight exposure trial the average solar radiation was 1024.8 ± 80.6 μmol/m^2^s, the average relative humidity was 51.6 ± 1.4 %, the average temperature was 35.1 ± 0.7 °C and the air was still (wind speed was 0 km/h). Figure 6 shows the averages of heart rate, respiration rate and infrared traits over the three portions of the trial (baseline, sunlight exposure, and shade period), as well as the environment temperature. The weather parameters of solar radiation, relative humidity, wind speed, and temperature all remained stable throughout the trial. Heart rate averaged 72.8 ± 16, 69.5 ± 22, and 70.5 ± 14 beats/min during the baseline, sunlight exposure and shade period respectively. The respiration rate averaged 36.3 ± 7.3, 51.5 ± 13.4 and 48.9 ± 12.1 counts/min during the baseline, sunlight exposure and shade period respectively, with the highest respiration rate for the sunlight exposure period observed shortly after moving the cows into sunlight (Fig. 6). The values for all infrared imaging traits increased immediately following exposure to sunlight. About 7 min after moving the animals to the shade, values returned to approximately those observed during the baseline portion, while the respiration rate remained elevated (Fig. 6). Sunlight exposure caused the greatest change in body surface temperature at the right flank (changing from 38.1 to 41.4 °C) and smallest change at the eyeball (changing from 37.2 to 38.1 °C).

### Biological factors

#### Physical exercise trial

**Fig. 7:**
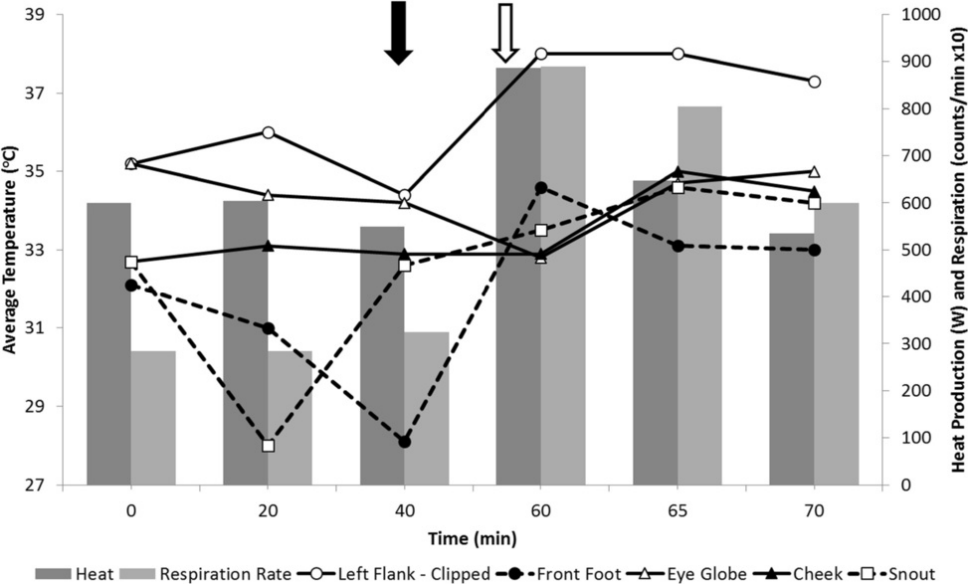
Infrared imaging measures, heat production and respiration rate in response to physical exercise. The *solid* and *open arrows* denote the start and end of the exercise challenge

The average relative humidity was 73.2 ± 2.7 % and the average temperature was 20.9 ± 0.1 °C during the physical exercise trial. The exercise trial revealed a noticeable increase in heat production and temperature for all body locations studied following the exercise treatment (Fig. 7). For example, the clipped left flank increased from 34.4 to 38.0 °C right after exercise. Respiration rate at baseline was 29.2 counts/min and increased to 80.1 counts/min immediately post-exercise. Changes in body surface temperature due to physical exercise were mirrored by heat production; the strongest association was observed with the snout, which increased in temperature from 32.6 to 33.5 °C when heat production increased. The weakest association was observed with the eyeball, which decreased in temperature from 34.2 to 32.8 °C as heat production increased (Fig. 7). At 5.0 min post-exercise, the increased heat production returned to values similar to those observed during the baseline assessment.

#### Drug response trial

**Fig. 8:**
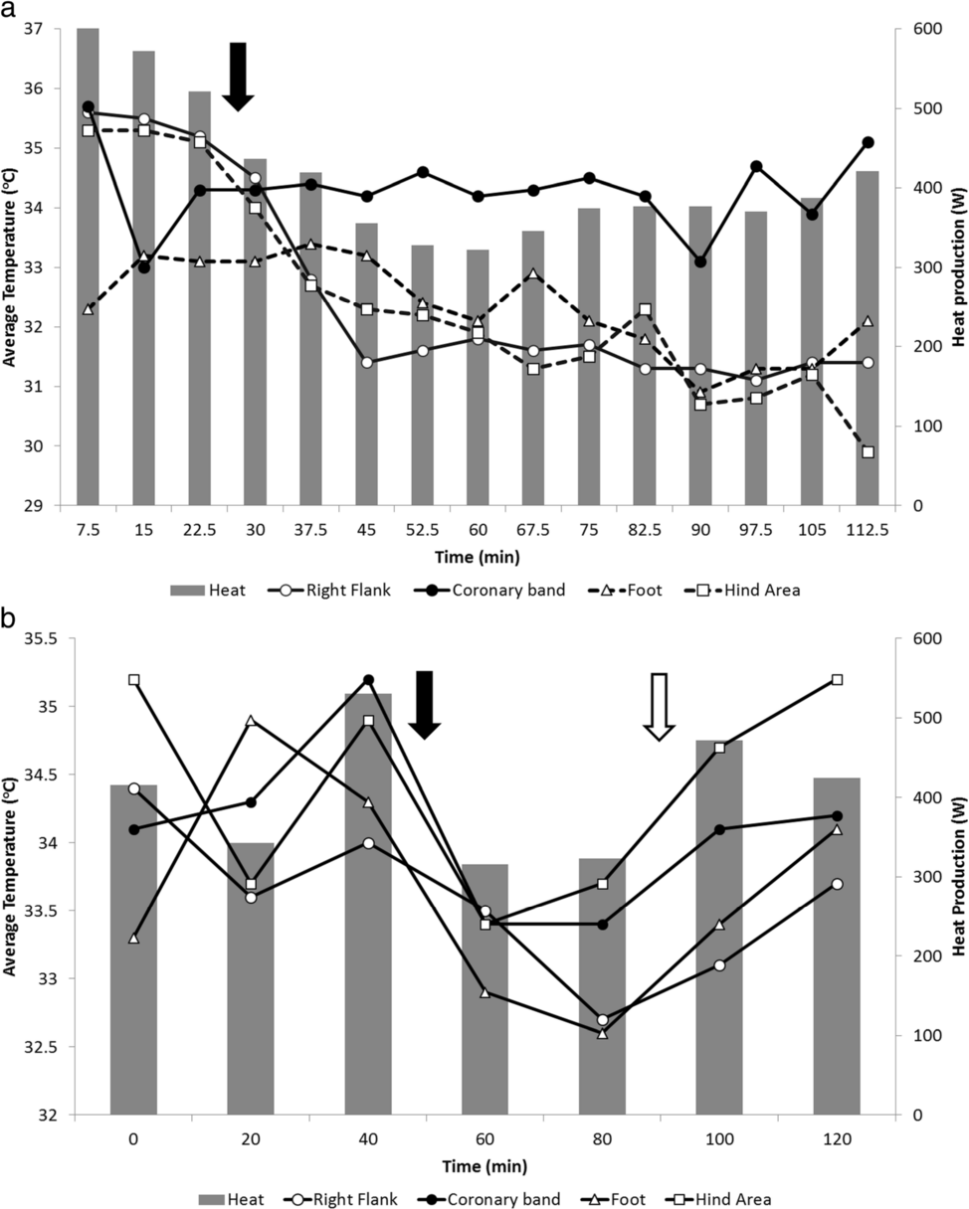
Infrared imaging measures and heat production in response to drugs. (**a**) Response to xylazine administration (only). (**b**) Response to xylazine followed by atipamezole administration. The *solid* and *open arrows* denote the injection of xylazine and atipamezole

During the conduction of the first and second portions of the drug trial, the average relative humidity was 73.8 ± 2.9 % and 74.2 ± 2.6 % and the average temperature was 23.9 ± 0.7 and 22.6 ± 0.6 °C, respectively. The first portion of the drug trial using xylazine only is illustrated in Fig. 8a. Heat production, upon administration of xylazine, decreased from 376.6 to 359.4 W. A greater decrease in heat production was observed in the second portion of the drug trial (Fig. 8b), where heat production decreased from 429.5 to 319.4 W with xylazine administration. Additionally, the effect of xylazine on metabolic rate was reversed by administration of atipamezole, as heat production values returned to values similar to those at baseline (Fig. 8b). Changes in body surface temperature due to drug administration were similar to the heat production pattern, with the strongest associations observed for the right flank and weakest for the coronary band (Fig. 8a, b).

#### Pregnancy status trial

**Fig. 9:**
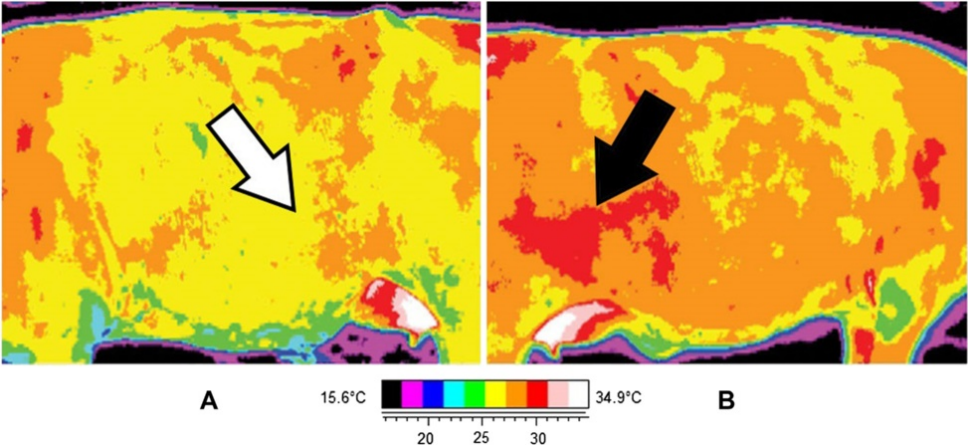
Infrared images of the left (**a**) and right (**b**) sides of the trunk of a pregnant cow. *Arrows* are pointing to the lower abdomen on both sides. The thermal print of the fetus on the right side is noticeable

Infrared assessment of pregnancy status was conducted at 20.4 °C and with a relative humidity of 54 %. Figure 9 shows images of the right and left sides of the Holstein heifer 2 d before calving. A noticeable temperature difference at the ventro-caudal portion of the trunk was observed when left and right sides were compared (Fig. 9a, b), with the right side having a well-delimited warmer region. The average temperatures on the warmer region and the adjacent region to this right side were 30.3 °C (maximum = 31.2 °C, minimum = 29.3 °C, SD = 0.5) and 28.7 °C (maximum = 29.4 °C, minimum = 27.1 °C, SD = 0.3), respectively.

## Discussion

### Technological factors

#### Imaging repeatability trial

As expected, the first study indicates a low association between infrared images taken on different days. This suggests that infrared imaging does not exhibit the strong repeatability of traditional routine practices employed within commercial (beef) cattle operations for assessing production characteristics, such as ultrasound for body composition [27, 28] and body weight [29]. This observation reflects the sensitivity of infrared imaging to metabolic state and environmental conditions, which would affect long term repeatability of other assessments (i.e. heart and respiration rate) rooted in the metabolic state. This lack of consistency was not observed when consecutive images (within 10 s) were taken; suggesting that constraints should be defined for proper infrared assessment over longer time periods. For example, factors such as ambient conditions [30] and familiarization with handling and management practices [31], were relatively uncontrolled in this study and may easily cause bias in the thermographs. The exposure of cattle to a several uncontrolled infrared imaging artifacts, including routine cattle handling and housing in commercial settings, may skew assessments by impairing regular radiation emission patterns from the body surface [32]. In essence, this initial study provides perspective for a series of eight studies aimed to further illustrate the boundaries of using infrared imaging for monitoring cattle in commercial operations.

#### Distance and infrared cameras comparison trial

Distance to the object is an influential factor on the results of an infrared imaging assessment due the effects on the captured radiation by infrared imaging devices [19]. Our results indicate an overall decrease in temperature (Fig. 2) as distance to the 5 by 5 cm shaven patch on the flank of the heifer increased (Fig. 1aj). This effect was due to the decreased number of pixels encompassing the targeted patch as a result of the increased distance, and was more prominent in the equipment with the lowest infrared imaging range (the i40; Fig. 2). Indeed, the fewer the pixels, as for camera i40 images, the lower the spot:size ratio that indicates the ability of an infrared camera to measure an object from a given distance [33]. Despite variation between infrared imaging technologies across different distances to the target (shaven patch), the observation that all the different equipment had consistent results for images taken up to 1.5 m from the target was remarkable. Such an observation indicates the possibility of using simpler technologies for performing infrared imaging in animals at close distances, which would be feasible in commercial operation set-ups (i.e. infrared devices mounted onto feeding stations), and improve economical reach and use of this technology. Similarly, infrared assessments at known distances to the object may aid in avoiding bias due to variation in distance and may be easily implemented with automated infrared imaging devices. Schaefer et al. [9] presented an intriguing example of an automated infrared imaging system which accommodated distance, resolution and other imaging issues to aid in early detection of an inflammatory respiratory disease in cattle at the feeding station.

#### Judge comparison trial

Infrared imaging evaluation is another key aspect of a promising thermographic assessment program. The current study demonstrated an outstanding agreement between analyses of thermographs made by two judges in six body locations, following a pre-established imaging protocol. The benefits of a pre-established infrared imaging analysis protocol were also demonstrated by Ammer [34] when comparing studies that analyzed the same body locations and concluded that variations of shapes and sizes of the measurement areas affected the precision of the body surface temperature readings. Similarly, a standardized landmark protocol established for capturing a series of thermographs over the entire human body yielded consistent results [1]. In contrast, analysis of magnetic resonance images compared across judges without a pre-defined imaging analysis protocol resulted in repeatability values of 0.4 [35]. This evidence reinforces the benefits of establishing imaging analysis protocols. Such protocols should be repeatable across judges and may eventually be incorporated within automated imaging analysis as part of a user-friendly technology for commercial operations.

### Environmental factors

#### Wind trial

Physical properties of heat loss in response to ambient conditions are important factors to consider when evaluating heat loss and body surface temperature patterns [36]. At the thermal neutral zone, heat production through radiation is at a maximum, increasing the capacity to make inferences on total heat production through detection of emitted radiation via infrared imaging. In our study, the room temperature was above the *Bos taurus* thermoneutral zone. For high-producing cattle with high intakes of metabolizable energy, such as those used in this study, the upper temperature limit may be closer to 20 °C [37]. However, the room temperature was still within the range where radiant heat loss is significantly important to regulate body temperature [38]. The wind caused an increase in convective heat loss that is primarily dependent on the movement of the air, which has been reported to affect body surface temperature in cattle [30]. Similarly, while studying heat loss in emu, Maloney & Dawson [39] observed that increased wind speeds decreased heat load from radiation. In our study, wind caused a cooling effect on the body surface that contradicted the close association between heat production and radiant heat captured by the infrared camera under still air conditions. Therefore, wind drafts that are a common event in handling facilities may mask the association between infrared measures and metabolic rate. The fact that the strong association between infrared imaging readings and metabolic rate was quickly re-established after ceasing the wind (Fig. 4) indicates that minimizing the effects of wind might be achieved through simple management adjustments and practices (i.e. wind breaks) at locations where the infrared images are taken.

#### Debris on body surface trial

Similar to the wind trial, the shavings affected body surface temperatures with no corresponding fluctuations in heat production. Interestingly, shavings resulted in a conductive cooling effect with heat being transferred from the higher temperature (animal) to lower temperatures (cooled shavings) through contact [36]. Even though heat loss through conduction is a minor contributor to the total heat loss in standing animals [40], these results indicate possible impairments in emitted radiation by the animal, at least temporarily. It is also important to highlight the fast return of an association between body surface temperature and heat production a few minutes after spreading cooled debris, indicating an area for optimizing handling practices. It is common to observe debris on cattle and there are studies demonstrating substantial effects of the amount of body coverage on thermoregulation and nutritional requirements [41]. Additionally, the type of debris should also be considered in relation to thermo-imaging properties. A study by van Liere [42] involving plumage in dust-bathing birds, found differences in temperature of the birds depending on the type of litter used for dust-bathing.

When the shavings were brushed off the animal there was a noticeable increase in the local temperature, which was unaccompanied by an increase in heat production (Fig. 5). It has been demonstrated that scratching causes an increase in peripheral blood flow, resulting in an increase in local heat dissipation [43]. Thus, our observation likely reflects this local heating generated by increased blood flow. This will have implications for infrared imaging in commercial set-ups, since cattle may rub their bodies during handling or as part of their general behavior. This can generate similar stimuli within the skin, resulting in unexpected and increased local heat dissipation inconsistent with metabolic state.

#### Sunlight exposure trial

The total infrared radiation captured through infrared imaging from an object represents the sum of emitted, transmitted and reflected radiation [39]. In the context of infrared imaging in animals, a combination of emitted plus reflected radiation is obtained when thermographs are taken under direct sunlight. In the present study, cattle were exposed to sunlight at both high humidity and ambient temperature. These factors combined altered the emitted radiation due to thermoregulatory adaptations by the animal, as demonstrated by the increase in respiration rate, indicating a greater heat loss as evaporative heat [44]. Interestingly, heart rate, a measure that can also indicate metabolic changes, was unaffected by sunlight exposure. As reviewed by Brosh [45], an increase in heart rate is expected in cattle kept under extreme and prolonged heat load, which is a more severe scenario than the conditions of our study.

Regarding changes in reflected radiation, moving the animal from shade to sunlight caused the body surface to reflect solar radiation in addition to the emitted radiation from the animal, both of which were captured in the thermographs. This resulted in a thermal imaging artifact where body surface temperature was apparently increased and subsequently decreased with sunlight and shade exposure respectively. Our study also indicated a rapid return to body surface temperatures similar to those observed during the baseline readings, upon 7 min of returning the cows to the shaded area. Similarly, dairy cows that chose shaded areas to rest were found to have decreased surface temperature after 10 min of staying in the shade [30]. It is also interesting to observe that the snout temperature decreased below the baseline readings after returning the cows to the shade. This biological response is probably due to changes in the peripheral vascularization patterns during heat dissipation, as illustrated elsewhere [26]. Considering the dramatic effects of solar loading on captured radiation from the body surface, this potential for bias should be considered in the thermography assessment of cattle subjected to sunlight exposure prior to infrared imaging. The reflected environmental radiation could also be largely accounted for, which is an alternative to minimizing the effects of solar load on the thermographs (i.e. by protocols and management prior to the imaging).

### Biological factors

#### Physical exercise trial

Physical exercise is known to produce an increase in metabolic rate [46], affecting the amount of heat produced and the means employed to dissipate heat [36]. Herein we note a substantial increase in heat production in response to exercise which is consistent with results observed by Folkow & Mercer [47] across different ambient temperatures and climate conditions. Similarly, elevated respiration rate in response to exercise is a known indicator of increased metabolic rate, as reported elsewhere [21, 48]. The association between heat production and infrared imaging measures in cattle is known [13], as well as the ability of body extremities to regulate body temperature [13, 26]. This evidence supports the close association observed between infrared measures of extremities and fluctuations in metabolic rate. Considering the tremendous variation in cattle temperament during handling [31] and the respective effects of exercising on metabolic rate, physical exercise can be considered a major bias while assessing metabolic rate. However, the observation that the metabolic rate returned to similar values as observed during the baseline readings indicates the potential for avoiding this confounding factor by standardizing the infrared imaging procedure in handled and restrained cattle.

#### Drug response trial

The decrease and re-establishment of body surface temperature and metabolic rate upon administration of a sedative and an anti-sedative were expected (Fig. 8a; b). Xylazine causes sedation and is associated with a hypothermic response in animals [49]. The duration of sedative effects we observed in the animals were comparable to other studies [50]. Conversely, atipamezole is known to provide a rapid reversal of xylazine-induced sedation and immobilization [51, 52]. Other studies have investigated the effects of drugs on the metabolic rate and skin temperature in the bovine [23], as well as the potential of anti-sedatives to reverse the metabolic effects of sedatives [53, 54]. Our study highlights the need to consider the significant effects of drugs on animal heat and temperature patterns while performing procedures that require chemical restriction, as well as use of infrared imaging in research models to evaluate the metabolic effects of administered drugs.

#### Pregnancy status trial

Pregnancy is a biological phenomenon that may be detected through infrared imaging due to the considerably higher metabolic rate of the fetus relative to that of the dam [55]. This discrepancy results in heat transfer by conduction through the tissue layers to the body surface, resulting in a comparatively warmer region detectable through infrared imaging. Remarkably, other studies have reported similar uses in a diversity of species such as giant pandas [56] and horses [22]. These studies, along with our own, indicate the potential for identifying pregnancy at its later stages, where the substantial metabolic output of the fetus is capable of producing enough heat to form thermal print. In our study, the observation of a thermal print on the right side of the cow likely indicated that the fetus was located within the right horn of the uterus, which may be a frequent occurrence [57, 58]. While pregnancy status may be predicted using infrared imaging, these observations suggest that the use of infrared imaging for pregnancy checks should be further evaluated to determine the earliest gestational stage for such an assessment. Jones et al. [59] conducted a preliminary study applying infrared imaging to detect pregnancy in dairy heifers and concluded that this form of assessment was confounded with ambient temperature and considered questionable; gestational stage, however, was not described.

## Conclusion

The three categories of factors influencing infrared imaging in cattle considered here illustrated both the occurrence of biases that can skew quality of the infrared imaging and possibilities for creating undesirable effects on infrared assessment. The use of such technology under relatively random conditions can result in misleading assessments, similar to the use of other biologically-sensitive measures. Indeed, the proper choice of equipment for a given application and imaging analyses plan, as well as the avoidance of environmental or biological biases, may maximize the successful application of infrared imaging in livestock production systems. Infrared imaging has tremendous potential for assessing aspects of bovine physiology related to health and productivity, which could ultimately bring benefits to the livestock industry.
